# The Bosadi-Salutogenic framework: culturally embedded identity as a personal resource for work engagement among Black South African women in middle management

**DOI:** 10.3389/fpsyg.2026.1832927

**Published:** 2026-10-13

**Authors:** Ongeziwe Gift Nodipha

**Affiliations:** Department of Industrial and Organizational Psychology, College of Economics and Management Sciences, University of South Africa, Pretoria, South Africa

**Keywords:** Afrocentric psychology, Black women, Bosadi, decolonization, job demands-resources model, salutogenesis, sense of coherence, work engagement

## Abstract

The dominance of Euro-American frameworks in industrial and organizational psychology has rendered culturally embedded work experiences largely invisible. While foundational models such as the Job Demands-Resources (JD-R) framework elegantly illuminate universal engagement mechanisms across diverse contexts, occupational health literature has traditionally paid less attention to how intersecting racialized and gendered identities shape occupational well-being in postcolonial settings. This conceptual paper addresses this contextual gap by examining the organizational position of Black women in South African middle management, a group navigating unique structural tensions rooted in historical exclusion and contemporary marginalization. Responding to calls for disciplinary decolonization, I propose an integrated Afrocentric framework that brings Bosadi theorization into conversation with salutogenic principles. Specifically, I argue that Bosadi identity functions are not merely as a demographic marker but as a culturally embedded personal resource that shapes how job demands are interpreted, managed, and made meaningful. By positioning identity as an analytic resource rather than a contextual variable, the framework extends the JD-R model’s explanatory power to account for culturally specific modes of resilience and meaning-making. The paper concludes by outlining theoretical contributions to organizational psychology and offering directions for empirical research, including methodological considerations for studying culturally situated work experiences. This work aims to support the development of organizational practices that are genuinely responsive to the lived realities of historically marginalized women.

## 1 Introduction

For decades, industrial and organizational psychology has established robust, empirically validated models of employee motivation and strain. The Job Demands-Resources (JD-R) framework exemplifies this scholarly achievement, demonstrating strong predictive validity across international workplace settings (Bakker and Demerouti, 2017; Schaufeli and Bakker, 2022). However, as organizational scholarship expands globally, researchers increasingly recognize the value of examining how deeply embedded cultural identities, historical legacies, and intersecting structural dynamics enrich these universal models (Buick, 2024; Rattrie, 2013). Rather than replacing established frameworks, incorporating indigenous and Afrocentric perspectives allows scholars to extend the boundary conditions of existing theories.

In South Africa, where apartheid’s spatial and economic logics continue to structure organizational life, this theoretical blind spot carries material consequences. In South Africa, where apartheid’s spatial and economic logics continue to structure organizational life, this theoretical blind spot carries material consequences for work engagement. Black women in middle management occupy a particularly complex position, navigating the daily dual burdens of racialized and gendered expectations while bearing responsibility for organizational delivery (Bowers, 2024; Carrim, 2019, 2021; Mngadi and Naidoo, 2023). Research indicates that Black South African women remain significantly underrepresented in management positions relative to their demographic representation, with multiple studies documenting experiences of subtle exclusion, microaggressions, and limited access to sponsorship networks (Carrim, 2019; Department of Employment and Labor, 2022; Human Rights Commission, 2023). These structural barriers are consistently associated with diminished work engagement reduced vigor, dedication, and absorption, yet the JD-R model provides limited tools for understanding how Black women sustain engagement despite such conditions. The model captures the demands these women encounter, workload, role ambiguity, and emotional labor, yet inadequately explains how they interpret, resist, or transform such demands through culturally specific meaning-making processes (Mokwena, 2023; Seyama-Mokhaneli and Belang, 2024).

This conceptual paper responds to that gap by advancing an integrated Afrocentric framework that brings Bosadi theorization into conversation with salutogenic principles. Drawing on African feminist scholarship (Masenya, 2005, 2020; Mokwena, 2023) and Antonovsky's (1979, 1987) concept of sense of coherence (SOC), I argue that Bosadi identity operates not merely as a demographic characteristic but as a culturally embedded personal resource that shapes engagement trajectories. The novelty of this integration lies in three contributions: (a) theorizing cultural identity itself as a personal resource within the JD-R framework, rather than treating it as a contextual variable or a source of demands; (b) demonstrating how Bosadi identity enriches the salutogenic concept of sense of coherence through culturally specific mechanisms; and (c) offering an operationalization of decolonial commitments within organizational psychology that centers African feminist knowledge systems as analytic resources. The intended audience includes organizational psychologists, human resource practitioners, diversity and inclusion specialists, and scholars of African feminism and decolonial studies.

Central to this argument is the recognition that work engagement among Bosadi-identifying women cannot be understood through frameworks that treat identity as peripheral to the core dynamics of engagement. The central problem this paper addresses is the theoretical invisibility of how culturally embedded identity sustains work engagement when conventional job resources are limited or absent. The framework repositions identity from a peripheral contextual variable to a central analytic resource, thereby extending the explanatory reach of occupational health psychology while contributing to ongoing efforts to decolonize the discipline. In what follows, I outline the theoretical foundations of this integration, develop a set of propositions to guide future empirical inquiry, and consider the implications for culturally responsive organizational practice in postcolonial contexts.

## 2 Theoretical foundations

This conceptual analysis was developed through an iterative process of literature synthesis, critical interrogation, and theoretical proposition development, following established methods for theory building in organizational psychology (Bakker and Demerouti, 2007; Bauer et al., 2019; Croswell and O'Connor, 2022).

### 2.1 Literature search and synthesis methodology

The theoretical foundations of this paper were assembled through a systematic review of literature across three intersecting domains: (1) Afrocentric psychology and Bosadi theorization, (2) salutogenic theory and the sense of coherence construct, and (3) engagement literature with specific attention to the JD-R model in non-Western contexts.

Searches were conducted using Scopus, Web of Science, Sabinet, and Google Scholar, covering publications from 1979 to 2026. Scopus and Web of Science were selected for their comprehensive coverage of peer-reviewed psychological literature; Sabinet was included to ensure coverage of South African and African-published scholarship; Google Scholar was used to identify grey literature and conference proceedings. The search was conducted in February 2026.

Inclusion criteria were: (a) peer-reviewed journal articles, books, or book chapters; (b) publications in English; (c) relevance to at least one of the three domains; (d) theoretical or empirical contributions to understanding identity, culture, and work. Exclusion criteria were: (a) publications focused exclusively on clinical or non-organizational populations; (b) studies conducted in contexts with no relevance to South African or African organizational settings; (c) publications lacking substantive theoretical or empirical contribution. The initial search yielded 847 records. After screening for relevance and duplicates, 142 sources were selected for full review. Of these, 94 were identified as foundational to the framework construction and are cited in this paper.

The theoretical synthesis proceeded through four stages: (1) identification of core constructs in each domain; (2) analysis of conceptual resonances and tensions across domains; (3) development of integrative propositions; and (4) construction of a visual model representing the integrated framework. This procedure follows established guidance for conceptual paper development in organizational psychology (Croswell and O'Connor, 2022; Jaakkola, 2020).

### 2.2 The Bosadi framework: cultural logic and identity formation

Black South African women’s experiences of work cannot be understood without reference to the intersecting histories of colonialism, apartheid, and patriarchy that have shaped their social positioning (Bowers, 2024; Carrim, 2019; Ratele, 2019). Under apartheid, Black women were subjected to systematic exclusion from formal employment, relegation to domestic and agricultural labor, and separation from their families through pass laws and migrant labor systems (Walker, 1990; Bonnin, 2001). Post-1994, while legislative frameworks changed, organizational cultures have been slower to transform. Importantly, Bosadi identity must not be conceptualized as a monolithic or essentializing construct. Black South African women represent a highly diverse demographic encompassing distinct ethnic heritage (including Zulu, Xhosa, Northern/Southern Sotho, Tswana, Swazi, Tsonga, Venda, and Ndebele), linguistic backgrounds, generational cohorts, geographic roots (urban, peri-urban, and rural), social classes, and faith traditions (Carrim, 2019; Mngadi and Naidoo, 2023). Bosadi does not imply an identical, uniform cultural orientation shared by all Black women; rather, it represents a rich, accessible cultural hermeneutic and resource repertoire that individual women negotiate, adopt, or modify in alignment with their personal agency and social positioning. It is within this context that Bosadi identity emerges as a significant cultural resource.

The Bosadi approach, as articulated by Masenya (2005, 2020), emerges from a deliberate epistemological rupture with Western feminist frameworks that have historically marginalized African women’s experiences. The term “Bosadi” transcends direct translation to encompass a distinctive mode of interpreting reality through the cultural lenses of Northern Sotho women. My analysis of Masenya’s foundational texts identifies four core principles structuring Bosadi identity, which I synthesize here to establish their relevance for organizational analysis.

- Cultural embeddedness positions identity formation within communal frameworks rather than individualistic paradigms. Mokwena (2023) observes that Bosadi reveals embedded cultural value construction that contrasts sharply with the autonomous self-theories underpinning conventional work engagement frameworks (Buick, 2024; Rattrie, 2013). For example, a Bosadi-identified woman in middle management may frame her career success not as individual achievement but as fulfilling obligations to her extended family, community, and ancestors. Decisions about career progression, relocation, or work-life balance are made in consultation with family elders and considered in light of communal impacts.
- Critical traditionalism characterizes the Bosadi approach as simultaneously affirming cultural heritage while problematizing patriarchal distortions within that heritage. Masenya (2020) demonstrates this dual movement by reading biblical texts alongside Northern Sotho proverbs, honoring indigenous knowledge systems while refusing to romanticize oppressive elements. In organizational terms, this might manifest as a woman drawing upon traditional values of community and collective responsibility while actively challenging gendered expectations that women should defer to male authority or sacrifice their careers for family obligations.
- Intersectional consciousness emerges from Black women’s location at the convergence of multiple marginalizations. Although Crenshaw (1989) formally theorized intersectionality within Black feminist legal scholarship, Masenya's (2005) earlier articulation demonstrates how African women’s hermeneutics independently recognized the compound effects of race, gender, class, and colonial history. This consciousness manifests in awareness that workplace challenges are shaped not only by gender but also by racial stereotyping, class-based assumptions about cultural competence, and the lingering effects of apartheid educational inequalities. A Bosadi woman reads workplace interactions through this layered interpretive lens.
- Epistemic agency positions Black women as knowledge producers rather than research objects. Masenya (2020) insists that Bosadi reading proceeds from the assumption that African women possess hermeneutical lenses capable of generating valid interpretations of text and the world. This principle informs the methodological approach of this paper: rather than treating Black women as subjects to be studied using external frameworks, the framework positions their cultural knowledge as a valid theoretical resource in its own right. In practice, this means that a Bosadi woman’s interpretation of workplace dynamics is treated as epistemologically valid rather than requiring validation through Western theoretical lenses.

Importantly, these principles are not uniformly experienced. Black South African women are diverse in ethnicity (Zulu, Xhosa, Sotho, Tswana, Tsonga, Venda, Ndebele, Swazi, and others), language, religious affiliation (including Christianity, African Traditional Religion, Islam, and others), generation, urban/rural background, and class position (Carrim, 2019; Mngadi and Naidoo, 2023). The Bosadi framework, while providing valuable interpretive resources, cannot be assumed to apply uniformly to all Black women. Future research must attend to this internal diversity.

### 2.3 Salutogenesis and sense of coherence: resources for meaning-making

Antonovsky's (1979, 1987, 1993, 1996) salutogenic model fundamentally reorients health research from pathogenesis toward understanding the origins of health and well-being (see also Mittelmark and Bauer, 2022). Rather than asking why people fall ill, salutogenesis asks how people maintain wellness under stress. The SOC represents the central construct mediating this orientation. Extensive systematic reviews have established the SOC as a valid and reliable construct across numerous studies (Eriksson and Lindstrom, 2005, 2006, 2007). However, these reviews also reveal a critical limitation: most research has been conducted in Western industrialized contexts, raising questions about how cultural frameworks shape the development and expression of SOC. Braun-Lewensohn and Sagy (2011) addressed this gap by demonstrating that adolescents from different cultural groups in Israel exhibited distinct patterns of personal and community SOC, suggesting that cultural context moderates salutogenic processes. This finding supports my argument that Black women’s SOC in South Africa may operate through culturally specific mechanisms not captured by standard measures.

The SOC comprises three interrelated dimensions:

*Comprehensibility* refers to the extent to which individuals perceive stimuli from internal and external environments as making cognitive sense, as ordered and consistent rather than chaotic (Antonovsky, 1987). However, what counts as “making sense” varies across cultural contexts. For Black women in South African organizations, comprehensibility may involve interpreting workplace events through frameworks that recognize systemic racism and sexism rather than individualizing these experiences.

*Manageability* denotes the degree to which individuals perceive resources as available to meet the demands posed by these stimuli (Antonovsky, 1987). Standard measures of manageability typically assess individual coping resources and social support networks. The Bosadi framework suggests that manageability for Black women may additionally involve communal resources—extended family obligations, church networks, and indigenous knowledge systems—that extend beyond conventional conceptualizations.

*Meaningfulness*, which Antonovsky (1987, p. 19) considered “the most crucial motivational component,” refers to the extent to which individuals feel that life makes emotional sense and that demands are challenges worthy of investment. Empirical research has consistently identified meaningfulness as the strongest predictor of health outcomes (Langeland et al., 2007; Vinje and Mittelmark, 2006). While Antonovsky emphasized existential and religious sources of meaning, the Bosadi framework suggests that meaningfulness for Black women may derive specifically from cultural identity, participation in ancestral traditions, responsibility to community flourishing, and connection to historical struggles for recognition (Mokwena, 2023; Seyama-Mokhaneli and Belang, 2024). Recent research in non-Western contexts supports this cultural specificity: Rajesh et al. (2015) found that SOC operates differently among Indian university students compared to Western samples, with community orientation playing a more prominent role.

### 2.4 The job demands-resources model: mechanisms of engagement

The JD-R model proposes that employee well-being emerges from the interplay between job demands and job resources (Bakker and Demerouti, 2007, 2017; Schaufeli and Bakker, 2022). Job demands constitute those physical, psychological, social, or organizational aspects of work that require sustained effort and therefore incur physiological and psychological costs. Job resources refer to those same aspects that reduce demands, facilitate goal achievement, or stimulate personal growth and development.

The model identifies two distinct psychological processes. The health impairment process occurs when excessive job demands exhaust employees’ mental and physical resources, leading to burnout over time. Conversely, the motivational process occurs when job resources satisfy basic human needs for autonomy, competence, and relatedness, thereby fostering work engagement characterized by vigor, dedication, and absorption. A third, less emphasized mechanism is the buffer effect, whereby job resources moderate the relationship between demands and strain: high resources buffer the negative impact of high demands, while low resources leave employees vulnerable to health impairment. In addition, the challenge-hindrance distinction suggests that not all demands are detrimental challenge demands (e.g., workload, time pressure) can stimulate growth and engagement when resources are adequate, whereas hindrance demands (e.g., role ambiguity, organizational politics) are consistently associated with negative outcomes (Crawford et al., 2010).

Despite substantial empirical support for the JD-R model, scholars increasingly recognize the need for culturally grounded extensions. Rattrie (2013) found that cultural factors moderate JD-R relationships, while Buick (2024) demonstrated that national culture influences antecedents of engagement. Recent work has incorporated contextual and cultural resources, including social support, organizational justice, and inclusive leadership (Bakker and Demerouti, 2017; Schaufeli and Bakker, 2022). However, these extensions have not fully addressed how culturally embedded identity itself functions as a personal resource. The claim that the JD-R model is rooted in individualistic worldviews should therefore be understood as a qualified observation: while the model has been extended to incorporate contextual factors, its core assumptions about the nature of personal resources remain individualistic (Mokwena, 2023; Seyama-Mokhaneli and Belang, 2024). Specifically, the JD-R model conceptualizes personal resources as individual psychological traits (e.g., self-efficacy, optimism, resilience) without adequately accounting for how cultural identity itself may function as a resource. The Bosadi-Salutogenic Framework addresses this limitation by theorizing culturally embedded identity as a personal resource that shapes both the perception of demands and the activation of resources.

### 2.5 Theoretical positioning of Bosadi as a personal resource

A central theoretical question must be addressed: Why should Bosadi identity be conceptualized as a personal resource within the JD-R framework rather than as a contextual resource, social resource, or merely a demographic variable? This section provides the theoretical justification for this positioning.

The JD-R model distinguishes between job resources (located in the work environment, e.g., autonomy, social support) and personal resources (individual characteristics that influence how employees interact with their environment, e.g., self-efficacy, optimism, resilience) (Bakker and Demerouti, 2017; Schaufeli and Bakker, 2022). Bosadi identity is theorized as a personal resource because it is internalized and carried by the individual across contexts it is not a feature of the job or organization but a characteristic of the person. However, unlike standard personal resources conceptualized as individual psychological traits, Bosadi identity is communal in origin and relational in expression. This paradox a personal resource with communal origins is precisely what makes Bosadi identity distinctive and theoretically interesting.

To clarify this distinction, consider existing constructs (Table 1):

**Table 1:** Key conceptual distinctions between established psychological constructs and Bosadi as a personal resource.

Construct	Definition	Key distinctions from Bosadi as a personal resource
Psychological capital (PsyCap)	Individual psychological capacity consisting of Hope, Efficacy, Resilience, and Optimism (Luthans et al., 2007).	PsyCap is conceptualized as state-like, individualistic, and universalist. Bosadi is socio-historically anchored, communal in origin, and functions as an active relational interpretive lens rather than an intrapsychic trait.
Social identity theory (SIT)	Self-concept derived from perceived membership in relevant social groups (Tajfel and Turner, 1979).	SIT primarily explains group categorization, in-group favoritism, and social comparison. Bosadi goes beyond group membership by providing a specific critical hermeneutic (*critical traditionalism*) that actively guides stress interpretation and resource activation.
Ethnic / Cultural identity	General sense of belonging, attachment, and pride in an ethnic or cultural group (Phinney, 1992).	General ethnic identity measures group attachment without specifying behavioral or cognitive coping mechanisms. Bosadi integrates explicit gendered, intersectional, and epistemic principles (*intersectional consciousness*, *epistemic agency*).
Collective efficacy	A group’s shared belief in its joint capability to organize and execute actions (Bandura, 1997).	Collective efficacy focuses on task-specific group competence. Bosadi operates as an individual-level personal resource with communal origins that provides existential meaning and structural sense-making

Social Identity Theory (Tajfel and Turner, 1979) posits that group membership provides individuals with a sense of belonging and self-worth. However, identity is typically treated as a background characteristic or source of bias, not as a resource that shapes engagement. Bosadi extends this by theorizing how cultural identity actively functions as a resource for interpreting demands, accessing support, and deriving meaning.

Conservation of Resources (COR) Theory (Hobfoll, 1989, 2001) proposes that individuals strive to retain, protect, and build resources. While COR theory acknowledges cultural resources, it does not specify how cultural identity operates within the JD-R’s engagement pathway. The Bosadi-Salutogenic Framework provides this specification by demonstrating the mechanisms through which Bosadi identity shapes comprehensibility, manageability, and meaningfulness. COR theory would suggest that Bosadi identity is a resource that individuals protect; the Bosadi-Salutogenic Framework extends this by showing how this resource is activated to foster engagement.

The framework does not claim that Bosadi identity functions identically for all women. As noted above, variation in ethnicity, generation, urban/rural background, and class position shapes how Bosadi identity is experienced and expressed.

## 3 The Bosadi-Salutogenic Framework: theoretical integration

Analysis of conceptual resonances across these theoretical domains reveals four points of integration that together constitute the Bosadi-Salutogenic Framework for work engagement among Black women in South African middle management (Figure 1).

**Figure 1:**
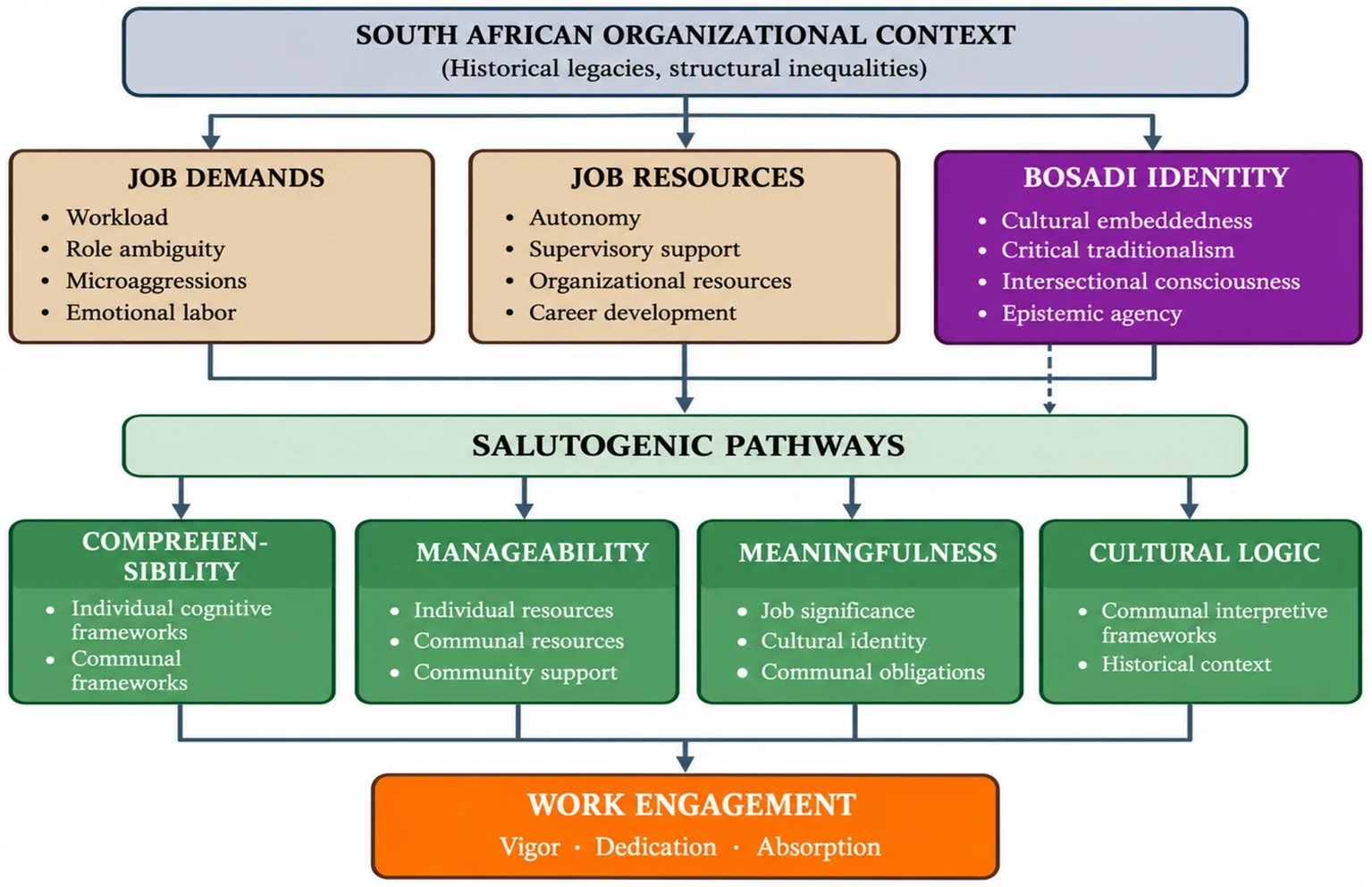
Conceptual model of the Bosadi-Salutogenic Framework. This figure illustrates the integrated theoretical framework. The model positions Bosadi identity as a central personal resource mediating between job demands/resources and engagement outcomes. Job demands (workload, role ambiguity, racialized and gendered microaggressions) and job resources (autonomy, supervisory support, organizational resources) are shown as inputs. Bosadi identity (comprising cultural embeddedness, critical traditionalism, intersectional consciousness, and epistemic agency) influences engagement through three pathways: comprehensibility (interpreting workplace stimuli through cultural frameworks), manageability (accessing communal resources), and meaningfulness (deriving purpose from cultural identity and communal obligations). The model is situated within the South African organizational context, recognizing the historical and structural factors shaping Black women’s experiences. Direct arrows indicate hypothesized positive relationships; the figure corresponds to Propositions 1-5.

### 3.1 Cultural logic as a comprehensibility structure

We propose that Bosadi identity acts as an interpretive filter through which workplace demands are processed. Where standard individualistic frameworks might frame workplace microaggressions primarily as interpersonal stressors requiring individual coping skills, a Bosadi-informed lens allows employees to recognize these dynamics as structural phenomena rooted in broader historical trajectories. Theoretically, this structural reframing is hypothesized to preserve individual self-worth and manageability by preventing the internalizing of systemic biases as personal inadequacies (Figure 2).

**Figure 2:**
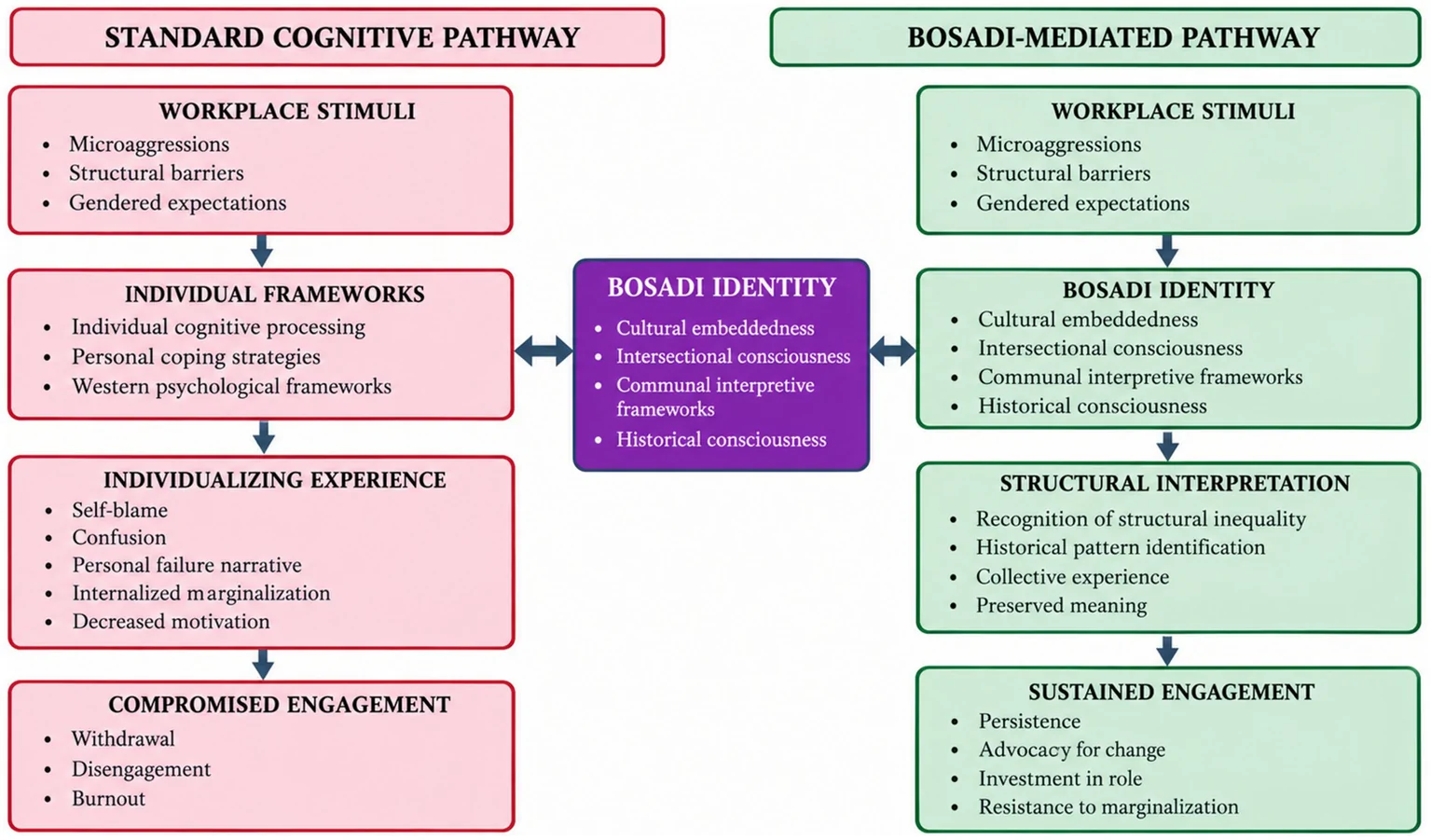
Bosadi identity as a comprehensibility structure. This figure illustrates the first integration point: cultural logic as a comprehensibility structure. The figure shows how Bosadi identity transforms the interpretation of workplace stimuli. In the standard cognitive pathway (left), workplace stimuli (microaggressions, structural barriers) are interpreted through individual frameworks, potentially leading to individualizing experiences (self-blame, confusion, personal failure). In the culturally mediated pathway (right), Bosadi identity provides communal interpretive frameworks that locate experiences within historical patterns of racialized and gendered exclusion. The outcome is a qualitatively different comprehensibility recognizing structural inequality rather than personal failure which preserves meaning and engagement. The figure demonstrates the alternative pathways for processing racialized and gendered experiences.

The first point of integration concerns how Bosadi identity functions as a culturally specific comprehensibility structure. For Black women navigating South African organizations, stimuli include not only generic work demands but also racially coded interactions and gendered microaggressions rooted in colonial and apartheid legacies (Bowers, 2024; Carrim, 2021; Mngadi and Naidoo, 2023). Bosadi identity provides interpretive frameworks for rendering these stimuli comprehensible.

Where Western psychological frameworks might interpret workplace microaggressions as isolated incidents requiring individual coping strategies, Bosadi frameworks locate them within historical patterns of racialized and gendered exclusion. This cultural reframing renders such experiences comprehensible not as personal failures or random misfortunes, but as manifestations of ongoing structural inequalities that Black women have historically navigated and resisted (Mokwena, 2023; Masenya, 2020). The comprehensibility achieved through Bosadi identity thus differs qualitatively from the cognitive sense-making described in standard SOC research. This reframing supports engagement by preserving meaning and motivation: when experiences are understood as structural rather than personal, women may be more likely to persist, advocate for change, and invest in their organizational roles rather than internalizing exclusion as evidence of inadequacy (Bowers, 2024; Seyama-Mokhaneli and Belang, 2024).

This integration extends Antonovsky's (1987) original formulation by demonstrating that comprehensibility is culturally mediated through shared interpretive frameworks. It must be noted, however, that not all Black women identify with Bosadi frameworks to the same degree. Urbanization, generational change, and religious conversion may influence the salience of Bosadi identity, with some women drawing on different cultural resources.

### 3.2 Communal resources and manageability

The second point of integration concerns how Bosadi identity expands the conceptualization of resources available for managing demands. The JD-R model traditionally conceives of job resources as located within organizational structures (e.g., supervisory support, autonomy) or individual psychological capacities (e.g., resilience, skills) (Bakker and Demerouti, 2017). Missing from this formulation are culturally embedded resources rooted in communal identity (Figure 3).

**Figure 3:**
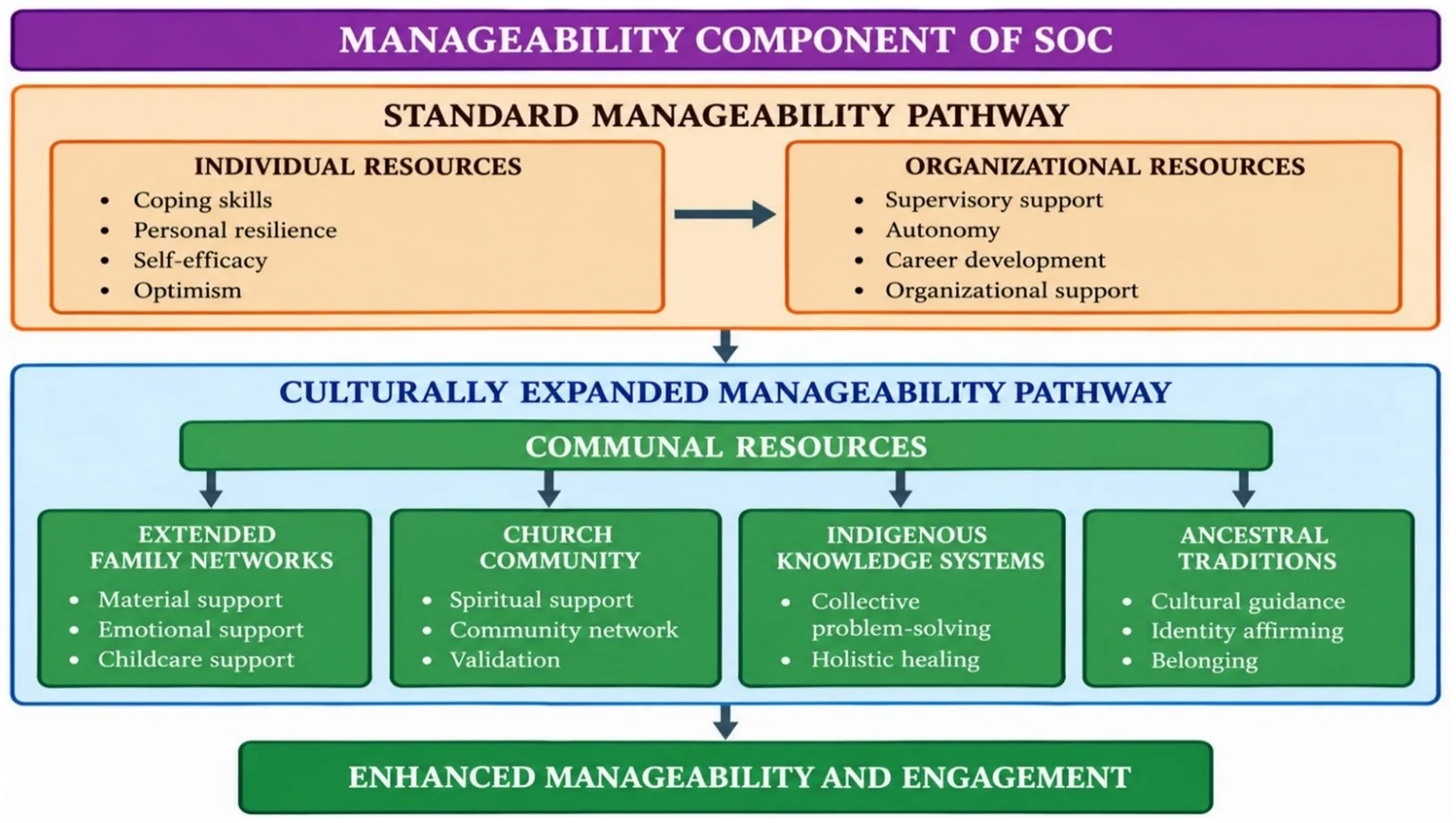
Communal resources expanding the manageability component of SOC. This figure illustrates the second integration point: how Bosadi identity expands the conceptualization of resources available for manageability. The figure contrasts the standard manageability pathway (top), which emphasizes individual coping resources and organizational support, with the culturally expanded pathway (bottom), which incorporates communal resources. These include extended family networks (material and emotional support), church communities (validation and meaning-making), indigenous knowledge systems (collective problem-solving and spiritual practices), and ancestral traditions (guidance and belonging). The figure demonstrates how Bosadi identity activates access to these resources and how they collectively enhance manageability. The model positions these communal resources as extensions of the JD-R model’s resource category.

Bosadi identity activates access to communal resources extending beyond the immediate workplace. For Black women in middle management, extended family networks provide material and emotional support that buffers against job stressors (Carrim, 2019; Mngadi and Naidoo, 2023). Church communities offer spaces for validation and meaning-making that counter workplace marginalization (Bowers, 2024; Ratele, 2019). Indigenous knowledge systems supply coping strategies such as collective problem-solving and spiritual practices that may not appear in Western psychological inventories (Seyama-Mokhaneli and Belang, 2024; Ndlovu-Gatsheni, 2018).

In addition to personal communal resources, workplace-based communal resources also play a role. Employee Resource Groups (ERGs), affinity groups, professional networks of Black women in management, and workplace community-building events can serve as organizational manifestations of communal support (Bowers, 2024; Mngadi and Naidoo, 2023). These workplace resources may be particularly valuable for women who lack access to personal communal networks or who wish to maintain boundaries between personal and professional life. The Bosadi-Salutogenic Framework recognizes both personal and workplace communal resources as pathways to enhanced manageability and engagement.

It is important to acknowledge that not all Black women have equal access to these communal resources. Women who have relocated for work, who are separated from family networks, who do not identify with formal religious institutions, or who have experienced community trauma may have limited communal resources available. For these women, workplace-based communal resources become particularly important. Additionally, some women may actively choose not to engage with communal resources due to personal history, religious conversion, or simply differing values. The framework does not assume universal access to communal resources; rather, it theorizes the mechanisms through which such resources, when available, enhance engagement. Future research should examine the conditions under which communal resources are most beneficial and the alternatives available to women who lack such resources.

Communal resources support work engagement through multiple mechanisms: (a) emotional buffering providing psychological safety and validation that counter workplace stressors; (b) practical support offering material assistance (childcare, transportation) that reduces the burden of job demands; (c) meaning reinforcement reminding women of the broader significance of their work beyond organizational metrics; (d) resistance solidarity providing spaces to share strategies for navigating exclusion and discrimination; and (e) identity affirmation reaffirming positive cultural identity in contexts where it is devalued (Carrim, 2019; Bowers, 2024; Mokwena, 2023).

This integration extends salutogenic theory by demonstrating that manageability is shaped by cultural frameworks that define what counts as a resource. For Bosadi-identified women, manageability may involve recognizing that one does not face demands alone, but as part of a network of reciprocal obligations and support that transcends organizational boundaries. This contrasts with the individualistic manageability emphasized in standard SOC research.

### 3.3 Meaningfulness and purpose grounded in Bosadi identity

The third and most significant point of integration concerns meaningfulness. The Bosadi framework reveals culturally specific sources of meaningfulness available to Black women that remain invisible within conventional occupational health psychology. To inhabit Bosadi womanhood is to participate in ancestral traditions and contribute to community flourishing (Mokwena, 2023; Masenya, 2020) (Figure 4).

**Figure 4:**
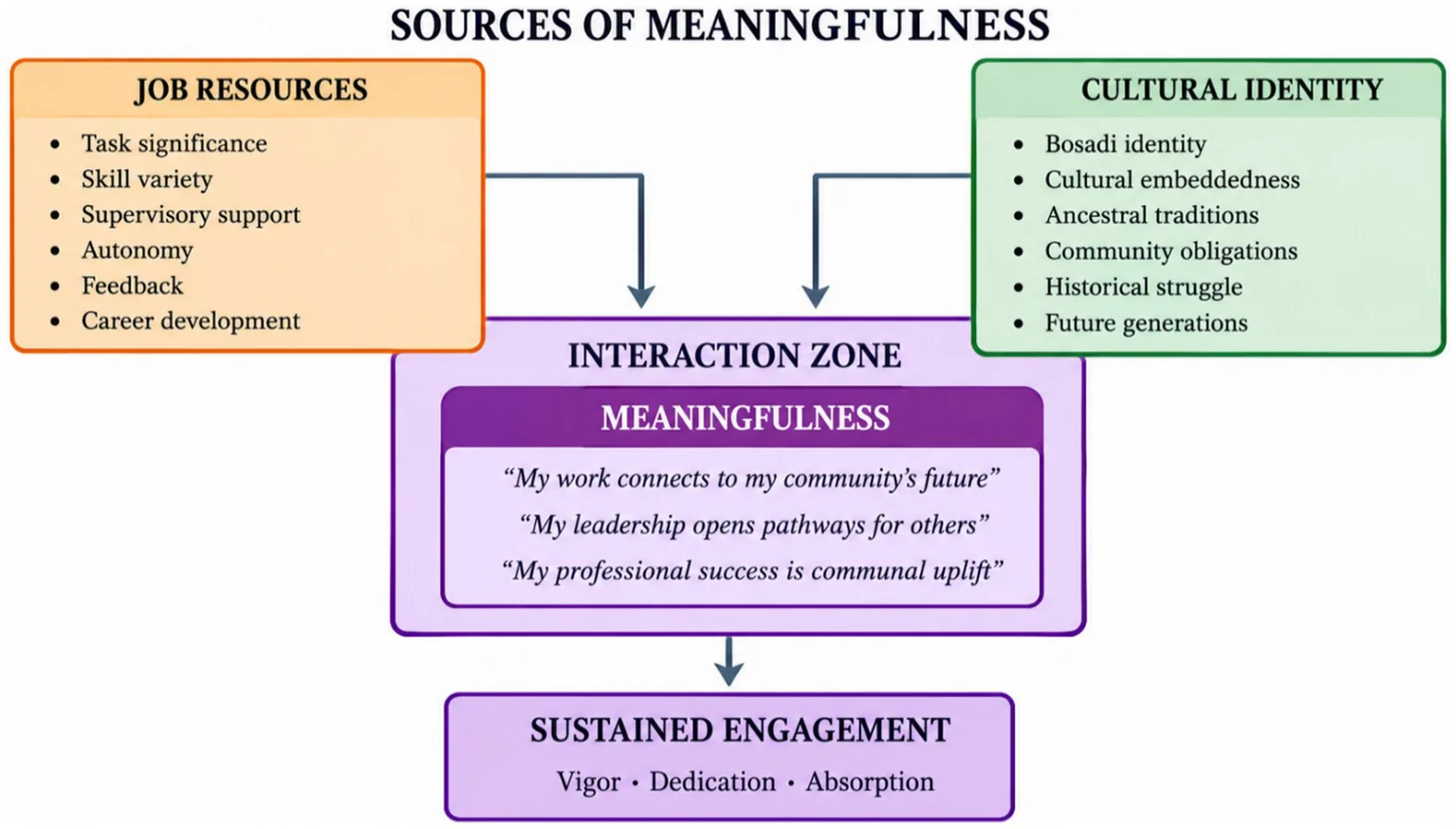
Sources of meaningfulness in the Bosadi-Salutogenic Framework. This figure illustrates the third integration point: meaningfulness grounded in Bosadi identity. The figure shows multiple sources of meaningfulness: job resources (task significance, skill variety, supervisory support) in the left panel; cultural identity and communal obligations in the right panel; and their interaction at the center. The arrows indicate how Bosadi identity provides a baseline of meaningfulness independent of job characteristics, how job resources contribute additional meaningfulness, and how they interact synergistically. The figure demonstrates that meaningfulness flows from cultural identity itself, which persists even when job characteristics are less than optimal.

For Black women in middle management, this culturally embedded meaningfulness transforms how job demands are experienced. Navigating racialized workplace dynamics constitutes participation in a longer historical struggle for Black women’s recognition and advancement (Bowers, 2024; Ratele, 2019). Leadership responsibilities carry meaning beyond organizational performance metrics, encompassing responsibilities to serve as role models and open pathways for those who follow (Seyama-Mokhaneli and Belang, 2024; Mngadi and Naidoo, 2023). Professional success thus connects to communal uplift rather than purely individual achievement.

This integration demonstrates that meaningfulness flows from cultural identity itself, independent of specific job design features. While the JD-R model emphasizes how job resources (e.g., task significance, skill variety) foster meaningfulness, the Bosadi-Salutogenic Framework suggests that cultural identity may provide a baseline of meaningfulness that persists even when job characteristics are less than optimal. This helps explain how Black women may maintain engagement in demanding organizational contexts that offer limited conventional resources. Importantly, this meaningfulness arises not only through spillover from personal to work life but also through within-work processes: the Bosadi woman may experience her professional role as an extension of her cultural responsibilities, making work itself a site of cultural expression and community contribution.

However, it is important to recognize that communal uplift has limits as a buffer. Research on resilience suggests that cultural resources are not infinite; sustained exposure to discrimination, excessive workload, and organizational exclusion can exhaust even the most robust cultural resources (Mngadi and Naidoo, 2023; Bowers, 2024). There is a threshold beyond which cultural meaning-making alone cannot compensate for material and structural barriers. The Bosadi-Salutogenic Framework does not propose cultural identity as a panacea for systemic inequality; rather, it theorizes identity as a resource that operates alongside not instead of structural interventions. When women lack basic organizational support, reasonable workloads, and fair treatment, even strong cultural identity may be insufficient to sustain engagement.

### 3.4 Intersectionality as an analytic resource

The fourth point of integration concerns how intersectionality functions within the proposed framework. Crenshaw's (1989) foundational articulation demonstrated that Black women’s experiences cannot be understood through additive models that treat race and gender as separate categories. South African scholarship has elaborated intersectionality’s relevance to organizational contexts (Carrim, 2019, 2021; Bowers, 2024; Mngadi and Naidoo, 2023) (Figure 5).

**Figure 5:**
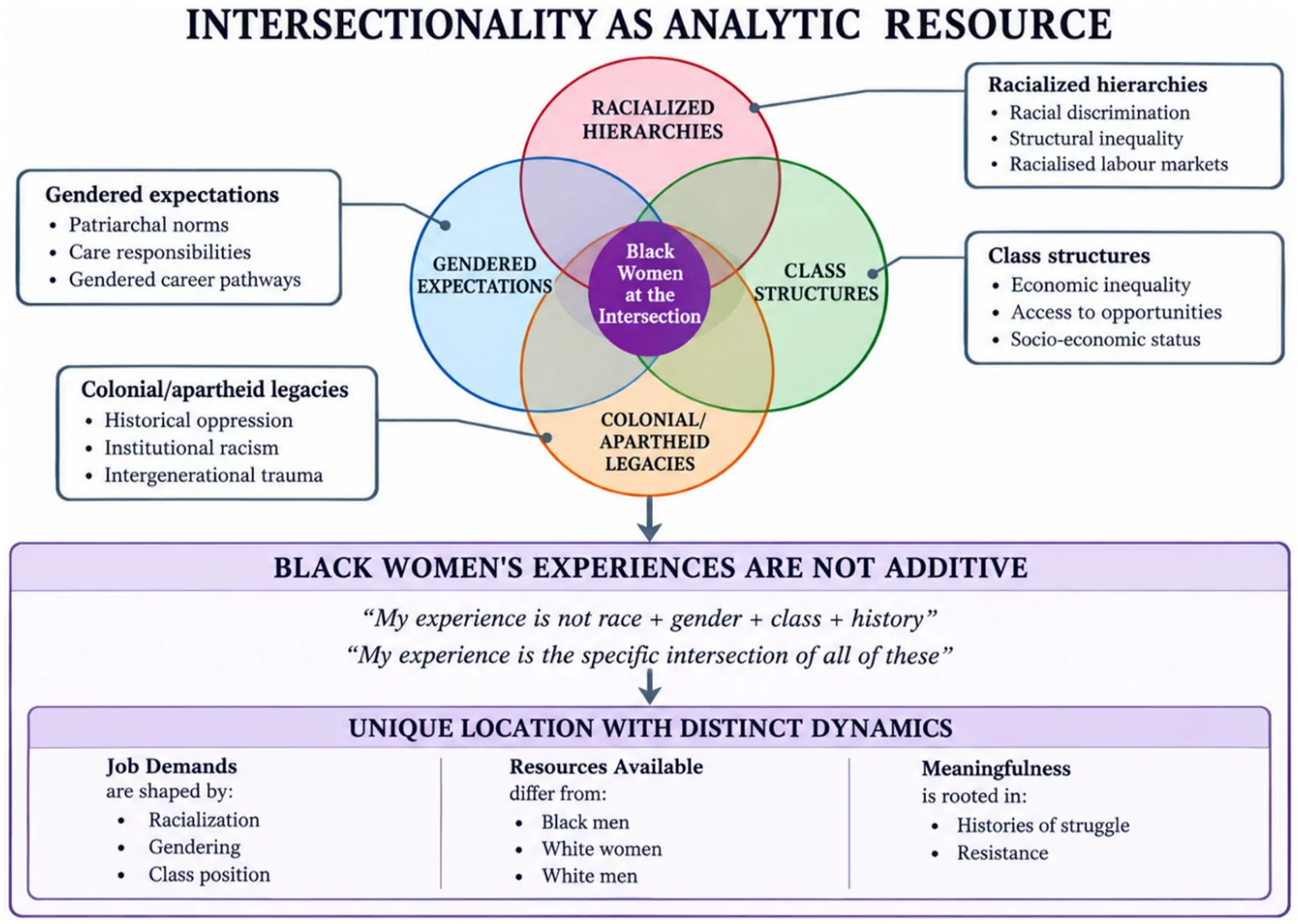
Intersectionality as an analytic resource in the Bosadi-Salutogenic Framework. This figure illustrates the fourth integration point: how intersectionality functions within the framework. The figure shows Black women positioned at the intersection of multiple systems of power racialized hierarchies, gendered expectations, class structures, and colonial/apartheid legacies. Each axis shapes job demands, resources available, and sources of meaningfulness. The figure demonstrates that experiences cannot be understood through additive models; instead, the specific intersection of multiple identities and structural positions constitutes a unique location with distinct dynamics. The dotted lines indicate how different intersections might apply to different women, reflecting diversity within the group.

Integrating intersectionality into the Bosadi-Salutogenic Framework ensures that analyses of work engagement attend to the specificity of Black women’s location. Job demands are shaped by simultaneous positioning within racialized hierarchies, gendered expectations, and class structures. Resources available to Black women may differ from those available to Black men or white women. Meaningfulness is rooted in the histories and struggles of Black women in postcolonial South Africa.

This integration prevents the framework from being appropriated as a generic “cultural” add-on to standard models. Instead, it insists that the Bosadi-Salutogenic Framework applies specifically to Black women’s experiences because these experiences are constituted at the intersection of multiple systems of power. The framework would require modification for application to other populations, not because culture is irrelevant to those populations, but because the specific cultural logic and intersectional location differ (Figure 6).

**Figure 6:**
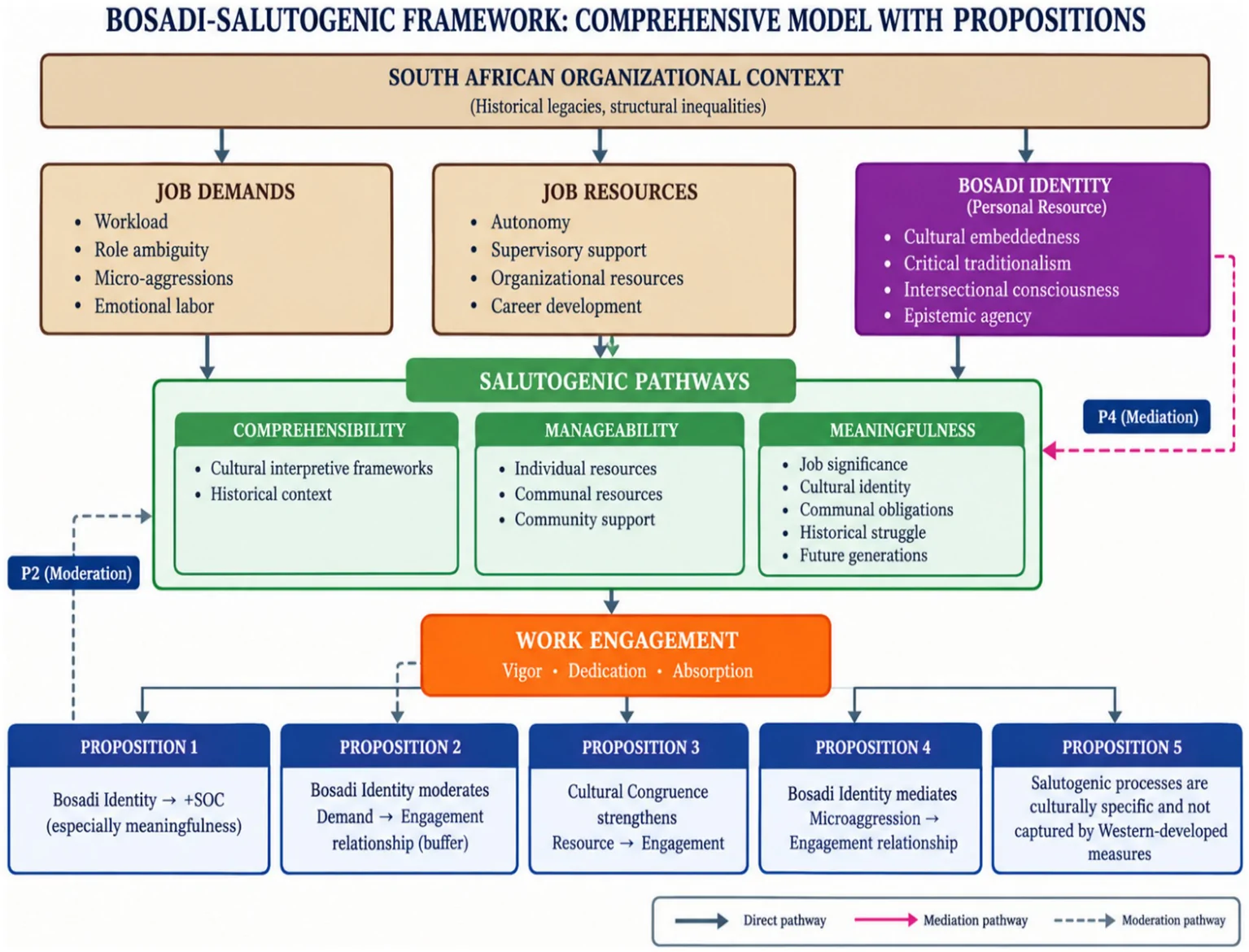
Comprehensive model with all propositions and hypothesized relationships. This figure presents the full integrated model with all five propositions. Each proposition is shown with its hypothesized relationships: Proposition 1 (Bosadi identity → higher SOC, especially meaningfulness), Proposition 2 (Bosadi identity moderates the demand-engagement relationship, with high-Bosadi women maintaining engagement at higher demand levels), Proposition 3 (resource-engagement relationship strengthened when resources are culturally congruent with Bosadi values), Proposition 4 (Bosadi identity mediates the relationship between microaggressions and engagement, reframing experiences through cultural lenses), and Proposition 5 (salutogenic processes are culturally specific, with Bosadi identity operating through mechanisms not captured by Western measures). Arrows indicate direct effects, dotted lines indicate moderating effects, and the mediation pathway is shown with specific relationships. The figure corresponds to the detailed propositions below.

### 3.5 Propositions for empirical testing

The conceptual integration yields five testable propositions:

Proposition 1: Bosadi identity and sense of coherence

Black women in South African middle management who report stronger identification with Bosadi cultural frameworks will demonstrate higher levels of sense of coherence, particularly on the meaningfulness dimension, compared to those with weaker Bosadi identifications.

*Rationale:* Bosadi identity provides interpretive frameworks, communal resources, and culturally grounded purpose that directly enhance the three SOC dimensions (comprehensibility, manageability, and especially meaningfulness). This proposition tests the central theoretical claim that culturally embedded identity functions as a salutogenic resource.

*Predictor:* Bosadi identity strength (measured by a culturally adapted Bosadi identification scale)

*Mediator:* Not applicable for this proposition

*Moderator:* Not applicable for this proposition

*Outcome:* Sense of coherence (with separate analysis of each dimension)

*Expected mechanism:* Cultural identity → enhanced comprehensibility through interpretive frameworks; enhanced manageability through communal resources; enhanced meaningfulness through culturally grounded purpose

Proposition 2: Bosadi identity as moderator of demands-engagement relationship

Bosadi identity moderates the relationship between job demands and engagement, such that high-Bosadi individuals maintain engagement at higher levels of demand than low-Bosadi individuals.

*Rationale:* If Bosadi identity functions as a resource that buffers against demands, the negative relationship between demands and engagement should be weaker for high-Bosadi women. This tests the resource-buffer hypothesis.

*Predictor:* Job demands (workload, role ambiguity, microaggressions)

*Moderator:* Bosadi identity strength

*Outcome:* Work engagement

*Expected mechanism:* Bosadi identity → reframing of demands as manageable and meaningful → sustained engagement despite demands

Proposition 3: Cultural congruence and resource-engagement relationship

The relationship between job resources and engagement is strengthened when resources are perceived as culturally congruent with Bosadi values.

*Rationale:* Resources that align with Bosadi values (community orientation, collective support, cultural affirmation) should be more effectively utilized than those that conflict with or are irrelevant to cultural values.

*Predictor:* Job resources (autonomy, support, development opportunities)

*Moderator:* Cultural congruence (perceived alignment with Bosadi values)

*Outcome:* Work engagement

*Expected mechanism:* Culturally congruent resources → activation of Bosadi identity → enhanced engagement

Proposition 4: Bosadi identity as mediator of microaggression-engagement relationship

Black women’s experiences of workplace microaggressions and subtle discrimination are mediated by Bosadi identity in their effects on engagement, with high-Bosadi women reframing such experiences through cultural lenses that preserve meaning and motivation.

*Rationale:* Bosadi identity provides interpretive frameworks that transform the meaning of microaggressions from personal failures to manifestations of structural inequality. These reframing preserves engagement.

*Predictor:* Workplace microaggressions and subtle discrimination

*Mediator:* Bosadi identity strength (particularly comprehensibility dimension)

*Moderator:* Not applicable for this proposition

*Outcome:* Work engagement

*Expected mechanism:* Microaggressions → Bosadi-mediated reframing (comprehensibility) → maintained or increased engagement

Proposition 5: Cultural specificity of salutogenic processes

The salutogenic processes linking Bosadi identity to engagement are culturally specific and cannot be fully captured by Western-developed measures of personal resources, psychological capital, or coping style.

*Rationale:* Standard measures assume individualistic conceptualizations of resources that may not capture communal resources, culturally specific meaning-making, or intersectional dynamics. This proposition tests the framework’s claim of cultural specificity.

*Predictor:* Bosadi identity strength (measured culturally)

*Mediator:* Not applicable for this proposition

*Moderator:* Cultural congruence of measures

*Outcome:* Work engagement

*Expected mechanism:* Bosadi identity → culturally specific salutogenic processes → engagement (not fully captured by standard Western measures)

## 4 Discussion

The Bosadi-Salutogenic Framework proposed in this conceptual paper offers a theoretical lens for understanding work engagement among Black women in South African middle management that centers culturally embedded identity as an analytic resource. The discussion interprets the framework’s implications, acknowledges limitations, and proposes directions for future research.

### 4.1 Extending the job demands-resources model

The proposed framework extends the JD-R model by identifying Bosadi identity as a culturally specific personal resource that shapes how job demands are interpreted and managed. Bakker and Demerouti's (2007, 2017) formulation, developed within Euro-American contexts, assumes a model of the self-autonomous and individualistic that does not universally apply (Buick, 2024; Rattrie, 2013). The Bosadi framework reveals a different ontology of the self: constituted through relationships rather than existing prior to them, embedded in communal history rather than self-created, and finding meaning through cultural participation rather than individual achievement (Mokwena, 2023; Masenya, 2020).

This extension aligns with Conservation of Resources (COR) Theory (Hobfoll, 1989, 2001), which recognizes that individuals draw upon multiple resource reservoirs personal, social, material, and cultural to prevent resource loss and promote resource gain. COR theory would predict that Black women invest in Bosadi identity as a resource to protect against workplace stressors, and that resource loss (e.g., through cultural erasure or exclusion) would be particularly damaging. However, COR theory does not specify how cultural identity operates within the JD-R’s engagement pathway. The Bosadi-Salutogenic Framework provides this specification by demonstrating the mechanisms of comprehensibility, manageability, and meaningfulness through which Bosadi identity fosters engagement.

Similarly, Social Identity Theory (Tajfel and Turner, 1979) suggests that group identification enhances self-esteem and belonging. However, standard applications of social identity in organizational psychology have treated identity as a source of in-group bias or adjustment, not as a resource for engagement. The Bosadi-Salutogenic Framework advances this understanding by theorizing how cultural identity actively functions as a resource that shapes engagement through specific psychological mechanisms.

Recent research on identity and engagement in non-Western contexts supports this argument. Daulat and Wadhwa (2023), studying Indian American women’s work engagement, found that identity self-acceptance contributed to positive mental health, which served as a personal resource for work engagement. Their findings, like those of the proposed framework, suggest that culturally specific identity processes are not merely contextual variables but active resources that shape engagement. Extending this line of inquiry, Daulat and Wadhwa (2025) conducted a qualitative exploration of South Asian American women’s ethnic-racial identity at work, demonstrating that wearing cultural dress (e.g., jhumkas and sarees) and engaging in cultural practices at work function as identity-affirming resources that support engagement and well-being. Their work provides empirical support for the argument that cultural identity operates as a personal resource within the JD-R framework, and that organizational contexts which allow for cultural expression foster greater engagement among women from minoritized backgrounds. These findings support the broader argument that JD-R research should expand conceptualizations of personal resources to include culturally embedded identity.

The framework does not reject the JD-R model’s core mechanisms but enriches them. Recent extensions of the JD-R model have incorporated contextual and cultural resources, including social support, organizational justice, and inclusive leadership (Bakker and Demerouti, 2017; Schaufeli and Bakker, 2022). However, these extensions have not fully addressed how culturally embedded identity itself functions as a personal resource. The Bosadi-Salutogenic Framework responds by theorizing the specific cultural mechanisms through which identity shapes engagement, moving beyond the observation that “culture matters” to specify how it matters for this population. The framework contributes to more recent JD-R scholarship by integrating this dimension.

### 4.2 Enriching salutogenic theory

The framework enriches salutogenic theory by demonstrating how sense of coherence is culturally mediated. Antonovsky (1987) acknowledged that SOC develops through life experiences shaped by social contexts, but his formulation left unspecified the mechanisms through which culture enters the salutogenic process. The Bosadi framework reveals specific mechanisms: comprehensibility achieved through communal interpretive frameworks, manageability assessed through awareness of communal resources, and meaningfulness derived from participation in historical struggles and communal purposes.

This enrichment suggests that salutogenic interventions should be culturally adapted rather than directly translated across contexts. For Black women in South Africa, strengthening SOC may involve reconnecting with cultural communities, engaging in indigenous meaning-making practices, and recognizing workplace struggles as connected to historical resistance. Standard cognitive-behavioral approaches to enhancing comprehensibility or individual skills training for manageability may miss these culturally specific pathways.

The framework also suggests that SOC may operate differently across cultural groups. The relative importance of the three SOC dimensions may vary, with meaningfulness potentially playing a more central role in collectivist cultural contexts. Future cross-cultural salutogenic research should examine these possibilities rather than assuming universal patterns.

### 4.3 Operationalizing decolonial commitments

The framework operationalizes decolonial commitments within organizational psychology by centering African feminist knowledge systems as analytic resources. Bharte and Mishra (2023) argued that decolonizing psychology requires fundamental rethinking of what counts as knowledge. The Bosadi-Salutogenic Framework responds by positioning Masenya's (2005, 2020) hermeneutic as a theoretical lens, refusing the extractive relationship characteristic of much cross-cultural research.

This operationalization involves several specific practices. First, the framework treats Bosadi not as a “local” variant of universal feminist theory, but as a distinct epistemological framework with its own integrity. Second, it positions Black women as theorists of their own experience rather than as data sources for Western theory testing. Third, it generates propositions that require methodologically innovative approaches such as yarning circles or narrative methods—rather than forcing Black women’s experiences into standardized Western measures.

The framework thus contributes to decolonization not only through its content but through its form. It demonstrates that rigorous organizational scholarship can emerge from engagement with indigenous knowledge systems, challenging the hierarchical relationship between “mainstream” (Western) and “critical” (non-Western) psychologies.

### 4.4 Practical implications

The framework suggests several principles for organizational practice responsive to Black women’s culturally embedded experiences.

#### 4.4.1 HR policy and diversity management

Organizations should recognize Bosadi identity as a resource rather than a liability. Current diversity practices often frame Black women’s cultural identities as sources of “bias” or “communication barriers” requiring assimilation to dominant norms. The Bosadi-Salutogenic Framework suggests instead that cultural identity provides resilience resources that organizations should actively support. Specific policy recommendations include: (a) revising dress codes and appearance policies to accommodate culturally significant practices; (b) recognizing cultural and religious holidays in leave policies; (c) developing flexible work arrangements that accommodate communal obligations; and (d) ensuring that diversity metrics track not only representation but also retention and progression of Bosadi-identified women.

#### 4.4.2 Leadership development and coaching

Leadership development programs should incorporate Bosadi perspectives by creating spaces where Black women can explore how their cultural identities inform their leadership philosophies (Seyama-Mokhaneli and Belang, 2024). This differs from standard leadership training that often requires participants to adopt predetermined leadership styles. Instead, Bosadi-informed development would support Black women in articulating leadership approaches grounded in their own cultural values. In coaching practice, this might involve: (a) using culturally grounded reflection tools that draw on indigenous knowledge; (b) connecting leadership development to community impact; (c) supporting coaches in navigating cultural dynamics in organizational contexts; and (d) creating peer coaching circles for Black women managers.

#### 4.4.3 Organizational inclusion programmes

Organizations should design resources accessible through communal channels. Rather than assuming that support must be individually delivered and privately received, organizations might create mentorship programs structured to honor communal values group mentoring, community celebration of achievements, or connections to cultural networks. Employee assistance programs might incorporate indigenous healing practices alongside conventional counseling. Specific Bosadi-informed inclusion initiatives could include: (a) establishing ERGs with dedicated resources and executive sponsorship; (b) creating structured networks that connect Black women managers across sectors; (c) recognizing communal achievements as part of performance evaluation; and (d) integrating African cultural practices (e.g., Ubuntu principles) into organizational values.

#### 4.4.4 Well-being interventions

Organizations should develop culturally sensitive approaches to well-being that explicitly connect organizational purposes to communal well-being. For Black women, work may be most engaging when they can see its connection to community flourishing. Organizations might articulate how individual roles contribute to broader social goods, create opportunities for community service, and support Black women in their roles as community leaders. Specific well-being recommendations include: (a) culturally adapted stress management programs; (b) access to culturally congruent mental health support; (c) recognition of community service as professional development; and (d) organizational support for women’s roles in community leadership.

### 4.5 Limitations and future research directions

The most significant limitation of this paper is its conceptual nature. The Bosadi-Salutogenic Framework requires empirical testing through methodologies appropriate to its theoretical commitments. Several specific limitations warrant acknowledgment.

First, the framework’s specificity to Black women in South African middle management limits its generalizability. The framework was developed for this specific population, and while it offers a template for similar culturally grounded extensions with other populations, direct transfer without adaptation would be inappropriate.

Second, confirmation bias is a potential limitation of any conceptual paper. The selection and interpretation of literature may reflect the author’s theoretical commitments. While I have attempted to critically engage with the literature, including challenges to the JD-R model’s universality, the framework would benefit from empirical testing and critical engagement from other scholars.

Third, conceptual subjectivity is inherent in this mode of theorizing. Different scholars might interpret Bosadi principles differently or identify additional integration points. The framework should be understood as a contribution to an ongoing conversation rather than a definitive statement.

Fourth, the framework risks essentializing Black women’s experiences if applied without attention to internal diversity. As noted throughout, Black South African women are heterogeneous by ethnicity, language, religion, generation, urban/rural background, and class. The framework does not apply uniformly, and future research must attend to this diversity. Boundary conditions include: (a) the framework applies most directly to women who identify with Bosadi cultural frameworks; (b) it is less applicable to women who have adopted alternative cultural orientations; (c) it applies specifically to middle management and may require adaptation for different organizational levels; (d) it assumes South African organizational contexts and may require modification for other national contexts; and (e) its applicability may vary by generation, with younger women potentially having different relationships to Bosadi identity.

Fifth, the framework emphasizes positive processes (engagement, resilience) and may insufficiently address the very real barriers and traumas Black women experience in South African organizations. The framework’s emphasis on cultural identity should not obscure the material conditions of pay equity, promotion opportunities, and safe working conditions that fundamentally shape occupational well-being. Cultural resources are not a substitute for structural change.

To advance the Bosadi-Salutogenic Framework from a conceptual model to an empirically testable paradigm, future research must address key operationalization challenges:

- Operationalizing Bosadi as a measurable construct: Researchers should undertake scale development to construct and validate the *Bosadi Personal Resource Scale (BPRS)*. Item generation should align with the four theoretical pillars: (a) *Cultural Embeddedness* (e.g., ‘My cultural heritage provides a foundation for how I approach professional challenges’); (b) *Critical Traditionalism* (e.g., ‘I draw strength from my cultural values while actively rejecting patriarchal workplace constraints’); (c) *Intersectional Agency* (e.g., ‘I view my unique identity as a source of insight in decision-making’); and (d) *Communal Support Activation* (e.g., ‘I draw on my community and network to navigate workplace stress’).
- Psychometric & discriminant validation: Psychometric evaluation utilizing Confirmatory Factor Analysis (CFA) and Structural Equation Modeling (SEM) is recommended to confirm construct validity. Discriminant validity must be rigorously demonstrated by establishing that the BPRS accounts for unique variance in work engagement (vigor, dedication, absorption) over and above established constructs, including Luthans’ PsyCap (PCQ-24), Phinney’s Multigroup Ethnic Identity Measure (MEIM), and Antonovsky’s Sense of Coherence Scale (SOC-13).
- Testing moderation and mediation models: Empirical testing of Propositions 1–5 should utilize multi-wave or daily diary designs to capture how Bosadi identity moderates the daily impact of racialized/gendered job demands on engagement. Structural equation modeling can test whether Sense of Coherence (specifically the meaningfulness dimension) acts as a mediator between Bosadi identity resources and work engagement outcomes.
- Methodological alignment: Given the decolonial and Afrocentric commitments of the framework, quantitative scale administration should ideally be paired with culturally congruent qualitative methods (e.g., conversational yarning, storytelling circles, or qualitative diary studies) to capture the depth of lived experiences without flattening cultural complexity.

## 5 Conclusion

The Bosadi-Salutogenic Framework offers a theoretical lens for understanding work engagement among Black women in South African middle management that centers culturally embedded identity as an analytic resource. By integrating Bosadi theorization with salutogenic principles and extending the JD-R model, the framework illuminates how cultural identity shapes the interpretation of job demands, the activation of resources, and the experience of meaningfulness at work.

Specifically, the framework demonstrates that cultural identity shapes interpretation in the following ways: (a) racialized and gendered workplace experiences are reframed from personal failures to manifestations of structural inequality, preserving meaning and motivation; (b) job demands are made manageable through access to communal resources (family, church, indigenous networks) that extend beyond individual coping; (c) professional work is infused with purpose derived from communal obligations, historical struggle, and responsibility to future generations; and (d) work engagement is sustained not only by organizational resources but by culturally grounded identity itself. The framework thus offers an alternative to the individualistic assumptions of standard models, positioning Black women as active meaning-makers rather than passive reactors to workplace conditions.

The framework contributes to theory development in occupational health psychology by expanding conceptualizations of personal resources to include communal assets, by demonstrating cultural mediation of sense of coherence, and by operationalizing decolonial commitments within organizational scholarship. These contributions address long-standing gaps in literature while maintaining rigorous theoretical standards.

Empirical testing of the framework’s propositions remains essential, requiring methodologically innovative approaches capable of capturing culturally specific phenomena without reducing them to Western categories. Such research should be conducted in partnership with Black women communities, honoring the epistemic agency that the Bosadi framework emphasizes.

Ultimately, the framework aspires to contribute to the development of organizational psychology adequate to cultural diversity and is committed to supporting flourishing across the full range of human cultural variation. By taking seriously the experiences of those historically marginalized by the discipline, the framework seeks not only to expand theoretical understanding but to support organizational practices that enable Black women to thrive as whole persons embedded in community, grounded in culture, and engaged in meaningful work.

## References

[ref1] Antonovsky A. (1979). Health, Stress and Coping. San Francisco, CA: Jossey-Bass.

[ref2] Antonovsky A. (1987). Unraveling the Mystery of Health: How People Manage Stress and stay well. San Francisco, CA: Jossey-Bass.

[ref3] Antonovsky A. (1993). The structure and properties of the sense of coherence scale. Soc. Sci. Med. 36, 725–733. doi: 10.1016/0277-9536(93)90033-Z, 8480217

[ref4] Antonovsky A. (1996). The salutogenic model as a theory to guide health promotion. Health Promot. Int. 11, 11–18. doi: 10.1093/heapro/11.1.11

[ref5] Bakker A. B. Demerouti E. (2007). The job demands-resources model: state of the art. J. Manager. Psychol. 22, 309–328. doi: 10.1108/02683940710733115

[ref6] Bakker A. B. Demerouti E. (2017). Job demands-resources theory: taking stock and looking forward. J. Occup. Health Psychol. 22, 273–285. doi: 10.1037/ocp0000056, 27732008

[ref9001] Bandura A. (1997). Self-Efficacy: The Exercise of Control. New York: W. H. Freeman and Company.

[ref7] Bauer G. F. Roy M. Bakibinga P. Contu P. Downe S. Eriksson M. . (2019). Future directions for the concept of salutogenesis: a position article. Health Promot. Int. 35, 187–195. doi: 10.1093/heapro/daz057, 31219568

[ref8] Bharte U. L. Mishra A. K. (2023). Working through the politics of indigeneity: decolonising psychology by way of a dialectical approach. Psychol. Dev. Soc. 35, 22–42. doi: 10.1177/09713336231154728

[ref9] Bonnin D. (2001). The state, gender and 'motherhood' in South Africa. Afr. Sociol. Rev. 5, 25–48.

[ref10] Bowers T. (2024). Developing an Intersectionality Framework for Exploring Gender Dynamics at Management level within a state-Owned Organization in South Africa. Cape Town, South Africa: University of Cape Town.

[ref11] Braun-Lewensohn O. Sagy S. (2011). Salutogenesis and culture: personal and community sense of coherence among adolescents belonging to three different cultural groups. Int. Rev. Psychiatry 23, 533–541. doi: 10.3109/09540261.2011.637312, 22272591

[ref12] Buick A. (2024). National culture has an impact on antecedents of engagement. Or does it? South Asian J. Bus. Stud. 13, 463–481. doi: 10.1108/SAJBS-03-2023-0089

[ref13] Carrim N. M. H. (2019). Who am I?'—South African Indian Women Managers' Struggle for Identity: Escaping the Ubiquitous cage. Pretoria, South Africa: AOSIS/Van Schaik.

[ref14] Carrim N. M. H. (2021). The in-betweeners: racio-ethnic and masculine identity work of Indian male managers in the South African private sector. J. Men's Stud. 29, 164–183. doi: 10.1177/1060826521993898

[ref15] Crawford E. R. LePine J. A. Rich B. L. (2010). Linking job demands and resources to employee engagement and burnout: a theoretical extension and meta-analytic test. J. Appl. Psychol. 95, 834–848. doi: 10.1037/a0019364, 20836586

[ref16] Crenshaw K. (1989). Demarginalizing the intersection of race and sex: a Black feminist critique of antidiscrimination doctrine, feminist theory and antiracist politics. Univ. Chicago Legal Forum 1989, 139–167.

[ref17] Croswell A. O'Connor P. (2022). Examining building blocks of well-being beyond PERMA and self-report bias. The Journal of Positive Psychology, 16, 811–818. doi: 10.1080/17439760.2020.1818813

[ref18] Daulat N. Wadhwa H. (2023). Examining Indian American women's relationship to work engagement through self-identity acceptance and positive mental health. Asian Am. J. Psychol. 14, 133–144. doi: 10.1037/aap0000279

[ref19] Daulat N. Wadhwa H. (2025). Wearing our jhumkas and sarees to work: a qualitative exploration of South Asian American women's ethnic-racial identity at work. Consult. Psychol. J. 77, 30–48. doi: 10.1037/cpb0000275

[ref20] Department of Employment and Labor (2022). Commission for Employment Equity Annual Report 2021–2022. Pretoria, South Africa: Government Printing Works.

[ref21] Eriksson M. Lindstrom B. (2005). Validity of Antonovsky's sense of coherence scale: a systematic review. J. Epidemiol. Community Health 59, 460–466. doi: 10.1136/jech.2003.018085, 15911640 PMC1757043

[ref22] Eriksson M. Lindstrom B. (2006). Antonovsky's sense of coherence scale and the relation with health: a systematic review. J. Epidemiol. Community Health 60, 376–381. doi: 10.1136/jech.2005.041616, 16614325 PMC2563977

[ref23] Eriksson M. Lindstrom B. (2007). Antonovsky's sense of coherence scale and its relation with quality of life: a systematic review. J. Epidemiol. Community Health 61, 938–944. doi: 10.1136/jech.2006.056028, 17933950 PMC2465600

[ref24] Hobfoll S. E. (1989). Conservation of resources: a new attempt at conceptualizing stress. Am. Psychol. 44, 513–524. doi: 10.1037/0003-066X.44.3.513, 2648906

[ref25] Hobfoll S. E. (2001). The influence of culture, community, and the nested-self in the stress process: advancing conservation of resources theory. Appl. Psychol. 50, 337–421. doi: 10.1111/1464-0597.00062

[ref26] Human Rights Commission (2023). Gender Transformation in the South African Workplace: A Report on progress and Challenges. Johannesburg, South Africa: South African Human Rights Commission.

[ref27] Jaakkola E. (2020). Designing conceptual articles: four approaches. AMS Rev. 10, 18–26. doi: 10.1007/s13162-020-00161-0

[ref28] Langeland E. Wahl A. K. Kristoffersen K. Nortvedt M. W. Hanestad B. R. (2007). Sense of coherence predicts change in life satisfaction among home-living residents in the community with mental health problems: a one-year follow-up study. Qual. Life Res. 16, 939–946. doi: 10.1007/s11136-006-9159-5, 17404897

[ref9002] Luthans F. Youssef C. M. Avolio B. J. (2007). Psychological Capital: Developing the Human Competitive Edge. Oxford: Oxford University Press.

[ref30] Masenya M. (2020). “Bosadi (womanhood) hermeneutics: re-reading the bible through African women's lenses,” in The Oxford Encyclopedia of the Bible and Gender Studies, ed. Dube M. W. (New York, United States: Oxford University Press), 87–103.

[ref29] Masenya M. J. (2005). Their hermeneutics was strange! Ours is a necessity! Rereading Vashti as African-South African women. In: Her master’s tools? Feminist and postcolonial engagements of historical-critical discourse. Eds. Vander Stichele C. Penner T.. Atlanta: Society of Biblical Literature. 175–194. doi: 10.1177/009182960503300205

[ref31] Mittelmark M. B. Bauer G. F. (2022). “Salutogenesis as a theory, as an orientation and as the sense of coherence,” in The Handbook of Salutogenesis, eds. Mittelmark M. B. Bauer G. F. Vaandrager L. Pelikan J. M. Sagy S. Eriksson M. . 2nd ed (Cham, Switzerland: Springer), 11–17. 36122001

[ref32] Mngadi N. Naidoo P. (2023). Black women in South African management: navigating intersectional identities. S. Afr. J. Labour Relat. 47, 112–130.

[ref33] Mokwena P. (2023). Mosadi ke motho: Madipoane Masenya's Bosadi approach as a critical framework for re-imagining gender from an indigenous African perspective. J. Gend. Relig. Afr. 29, 45–62.

[ref34] Ndlovu-Gatsheni S. J. (2018). Epistemic Freedom in Africa: Deprovincialization and Decolonization. London, United Kingdom: Routledge.

[ref9003] Phinney J. S. (1992). The Multigroup Ethnic Identity Measure: A new scale for use with diverse groups. Journal of Adolescent Research, 7, 156–176. doi: 10.1177/074355489272003

[ref35] Rajesh G. Eriksson M. Pai K. Seemanthini S. Naik D. G. Rao A. (2015). The validity and reliability of the sense of coherence scale among Indian university students. Glob. Health Promot. 23, 16–23. doi: 10.1177/1757975915601297 25897012

[ref36] Ratele K. (2019). The World Looks like this from here: Thoughts on African Psychology. Johannesburg, South Africa: Wits University Press.

[ref37] Rattrie L. T. B. (2013). The role of Demands and Resources in the International work context: Conceptual Approach and Empirical Analysis. Doctoral thesis. Stirling, United Kingdom: University of Stirling.

[ref38] Schaufeli W. B. Bakker A. B. (2022). “The job demands-resources model: a comprehensive review and future directions,” in Handbook on the temporal Dynamics of Organizational Behavior, eds. Perrewé P. L. Harms P. D. Chang C. H. (Cheltenham, United Kingdom: Edward Elgar), 123–145.

[ref39] Seyama-Mokhaneli S. Belang T. (2024). Decolonial identities in the leadership coaching space: against neoliberal leader identity regulation. Front. Psychol. 15:1380610. doi: 10.3389/fpsyg.2024.1380610, 38860042 PMC11164188

[ref40] Tajfel H. Turner J. C. (1979). “An integrative theory of intergroup conflict,” in The social Psychology of Intergroup Relations, eds. Austin W. G. Worchel S. (Monterey, CA: Brooks/Cole), 33–47.

[ref41] Vinje H. F. Mittelmark M. (2006). Deflecting the path to burn-out among community health nurses: how the effective practice of self-tuning renews job-engagement. Int. J. Ment. Health Promot. 8, 36–47. doi: 10.1080/14623730.2006.9721844

[ref42] Walker C. (ed.). (1990). Women and Resistance in South Africa. Cape Town, South Africa: David Philip; London, United Kingdom: James Currey.

